# Vitamin D and Vitamin D Analogues in Hemodialysis Patients: A Review of the Literature

**DOI:** 10.3390/ijms262311550

**Published:** 2025-11-28

**Authors:** Konstantia Kantartzi, Stefanos Roumeliotis, Christos Polychronidis, Elena Zafeiri, Athanasios Roumeliotis, Konstantinos Leivaditis, Vassilios Liakopoulos

**Affiliations:** 1 Department of Nephrology, University Hospital of Alexandroupolis, Democritus University of Thrace, 68100 Alexandroupolis, Greecech.polych@gmail.com (C.P.); zafeiri.el@gmail.com (E.Z.); 2 2nd Department of Nephrology, AHEPA University Hospital Medical School, Aristotle University of Thessaloniki, 54636 Thessaloniki, Greece; a_roumeliotis@hotmail.com (A.R.); vliak@auth.gr (V.L.)

**Keywords:** chronic kidney disease, hemodialysis, vitamin D

## Abstract

Vitamin D exists in various forms and plays a central role in the absorption and regulation of calcium and phosphate. In chronic kidney disease, vitamin D concentrations become progressively reduced with the deterioration of kidney function, which becomes even more pronounced in end-stage kidney disease. Herein, we aim to summarize existing data regarding the pathogenetic role of vitamin D in dialysis and the potential effect of supplementation of various forms of vitamin D on hard and surrogate clinical endpoints. We performed a narrative review, gathering existing observational and clinical studies from 2001 to 2025 in English in the Medline/PubMed database, along with current guidelines and consensus statements regarding the use of vitamin D and D analogues in end-stage kidney disease patients. Vitamin D should be monitored and corrected, but supraphysiologic doses should be avoided, as well as very high levels of vitamin D to avoid toxicity. In dialysis, native D is used only to correct vitamin D deficiency; the real target here is secondary hyperparathyroidism, where vitamin D analogues and calcimimetics should be administered.

## 1. Introduction

Chronic kidney disease (CKD) has reached pandemic proportions as its incidence and prevalence continue to increase worldwide. More than 10% of the population has CKD [1]. As kidney function declines, the physiological roles of the kidneys are impaired, leading to CKD complications, such as mineral and bone disorders (MBD), which become more severe and evident in end-stage kidney disease (ESKD), especially among hemodialysis (HD) patients. Vitamin D is a crucial hormone that plays a pivotal role in the regulation of calcium, phosphate, and bone mineral metabolism [2]. Active Vitamin D regulates the expression of nuclear vitamin D receptor (VDR) genes. VDR is a DNA-binding protein transcription factor recruiting active chromatin that might affect the transcription process [3]. Vitamin D includes a group of fat-soluble, steroid compounds that are essential for the absorption and management of phosphate and calcium. Vitamin D2 (also known as ergocalciferol) is synthesized by plants and mushrooms and can be obtained only through exogenous dietary sources or supplements. Cholecalciferol (also known as vitamin D3) is exogenously and endogenously produced though the photolysis of its precursor, 7-dehydrocholesterol, in the skin when exposed to sunlight [4]. Vitamin D metabolism in healthy subjects occurs in three basic steps. Initially, it is synthesized in the skin, then the liver converts the inactive form of vitamin D into 25(OH)D, which is further converted by the kidneys into the active form 1,25(OH)D_2_ by the enzyme 1-a-hydroxylase. Several factors might regulate the kidneys’ activation of vitamin D, including circulating calcium, phosphate, PTH, and FGF-23. The active vitamin D calcitriol plays a pivotal role in the regulation of phosphate and calcium and bone metabolism. Through activation of VDR, calcitriol triggers increased reabsorption of calcium and phosphate in the kidneys, increased calcium absorption from the intestine, and bone mineralization, leading eventually to the development of hypercalcemia and hyperphosphatemia.

To minimize these adverse renal and intestinal effects and still maintain optimal control of SHPT, natural and active forms of vitamin D have been modified or resynthesized to develop pharmaceutical agents with improved selective actions and properties. These vitamin D analogues (VDAs) include paracalcitol, doxercalciferol, and alfacalcidol, and they have different molecular structures than active vitamin D (side chains or other modifications), which allows them to bind selectively to VDR in the parathyroid glands and therefore reduce the risk of hypercalcemia and hyperphosphatemia while suppressing PTH levels in a dose-dependent manner. 

Although vitamin D improves several clinical outcomes, this might differ in healthy subjects and specific groups. In CKD, there are significant alterations in the metabolism of vitamin D as kidney function declines; therefore, screening for vitamin D deficiency, the roles of vitamin D, and recommendations for vitamin D administration vary significantly between predialysis CKD 1–4 stages and HD patients.

Although current nephrology guidelines suggest monitoring and correcting vitamin D deficiency in CKD quite similarly to the general population, recent large and well-designed clinical trials did not demonstrate any clear cut clinical benefit of vitamin D on skeletal and non-skeletal outcomes in CKD. Moreover, due to impaired kidney clearance, vitamin D in high dosages might cause toxicity. During the last three years, several meta-analyses, trials, and landmark studies have been published on CKD and ESKD populations; moreover, this year, a joint European consensus statement was published for CKD and ESKD patients. Herein, we aim to present and discuss all of these data regarding vitamin D in dialysis patients.

In this narrative review, we aim to summarize, discuss, and assess data from existing studies, consensuses, and guidelines.

## 2. Results

### 2.1. Vitamin D Forms and Their Use in Hemodialysis

KDIGO guidelines for ESKD patients undergoing maintenance HD recommend assessing calcidiol levels at baseline and once a year (or every 6 months if being treated for deficiency) and correcting vitamin D insufficiency or deficiency with therapeutic strategies that are commonly used for the general population, i.e., supplementation of D2 or D3 (2C level of recommendation) [5].

However, besides treating low vitamin D levels, the supplementation of native vitamin D in dialysis patients might not have beneficial effects on CKD-MBD or any clinical hard endpoints (including survival), and therefore the optimal approach for this population is an area of debate. This might be explained by the fact that in dialysis, the main target for treating CKD MBD is SHPT. VDAs might be of use in this context, as their potential benefits include improved bone health and reduced cardiovascular disease and mortality. Although reduced, the risks of hypercalcemia, hyperphosphatemia, and adynamic bone disease remain significant. The dosage of VDAs should be individualized and carefully monitored, and VDAs could be combined with calcimimetics to achieve optimal SHPT management [6].

### 2.2. Pleiotropic Actions of Vitamin D

Vitamin D is the main regulator of bone and muscle function, nerve conduction, and other cellular functions [7]. Because VDR and coenzyme CYP27B1 are found and expressed in various tissues and organs, vitamin D exerts pleiotropic, extraskeletal actions on cell proliferation, immunity, muscle and bone growth, skin differentiation, and cardiovascular health.

Proteinuria and CKD progression: Low circulating Vitamin D might trigger the onset and progression of both kidney disease and albuminuria, while VDR activation can prevent the progression of kidney disease. There is a growing body of evidence suggesting a renoprotective role of vitamin D administration in IgA nephropathy, [8] lupus nephritis [9], and diabetic kidney disease [8,9]

Autoimmunity, inflammation, and infection: Vitamin D modulates both T- and B-cell proliferation, stimulates anti-inflammatory cytokine production, suppresses inflammatory cytokine synthesis, and affects autophagy and apoptosis [10]. Through these mechanisms, vitamin D upregulates natural protective autoimmunity mechanisms. In HD, vitamin D insufficiency or deficiency might predispose to infections and hospitalizations, as repeatedly reported in these patients. Moreover, observational data suggest that supplementation with vitamin D analogues might reduce infection rates in this population. Tsujimoto et al. conducted a prospective study including 508 maintenance dialysis patients followed for 5 years and found that treatment with VDAs decreased the incidence of hospitalization due to acute infections of the respiratory system (hazard ratio (HR) = 0.47, 95% confidence interval (CI) 0.25–0.90) [11]. The linkage between vitamin D and infections in dialysis patients is thought to be even more pronounced in COVID-19 infections. Among maintenance dialysis patients, those who were positive for COVID-19 had marked vitamin D deficiency and received only low dosages of the VDA alfacalcidol [12]. However, other data reported no association between vitamin D concentrations and COVID-19 infection in HD populations. Moreover, it seems that vitamin D might play a crucial role in maintaining a sufficient concentration of anti-SARS-CoV-2 antibodies post-vaccination in HD patients [13], and active vitamin D might protect against life-threatening SARS-CoV2-derived pneumonia in HD patients [14]. Currently, existing data regarding the potential protective effect of vitamin D and VDAs in HD against COVID-19 are contraindicatory. Large, well-designed RCTs are needed in order to draw definite conclusions.

Cardiovascular disease (CVD): CKD is accompanied by increased morbidity and mortality, which are mainly attributed to CVD [15] and might be partially attributable to vitamin D deficiency. Vitamin D receptors are also expressed in cardiovascular sites, indicating a possible implication of vitamin D in cardiovascular health. VDR activation might protect cardiovascular health through anti-inflammatory effects and improvements in smooth muscle cell contractility, proliferation, and growth. An association between vitamin D deficiency and CVD has been reported in healthy subjects and in CKD, but large randomized intervention trials have failed to reveal positive effects in cardiovascular outcomes [16]. Restoration of normal levels of vitamin D failed to show any improvement in blood pressure, endothelial function, or arterial stiffness in uremic patients [17].

Cognitive function: Recent studies in CKD populations suggest that vitamin D deficiency might be associated with mental and cognitive function. Experimental data demonstrate that vitamin D is a necessary compound for both brain development in childhood and ongoing brain function in adults by affecting synaptic plasticity, neuronal protection, neural circuit formation, and the dopaminergic system [18]. This association is suggested to start in pregnancy and continue in offspring. Moreover, patients with neurodegenerative diseases (e.g., dementia, Alzheimer’s disease) and neuroinflammatory and neuropsychiatric conditions have significantly reduced vitamin D levels. However, restoration of vitamin D concentrations failed to show any improvement in dementia and cognitive dysfunction [19]. In CKD and ESKD, scientific interest has focused on early detection and therapeutic management of cognitive dysfunction. Currently, data on the effects of vitamin D on mental health in patients with CKD are very limited, with large heterogeneity, small sample sizes, and inconclusive results.

### 2.3. Reasons for Vitamin D Deficiency/Insufficiency in Dialysis Patients

**Figure 1:**
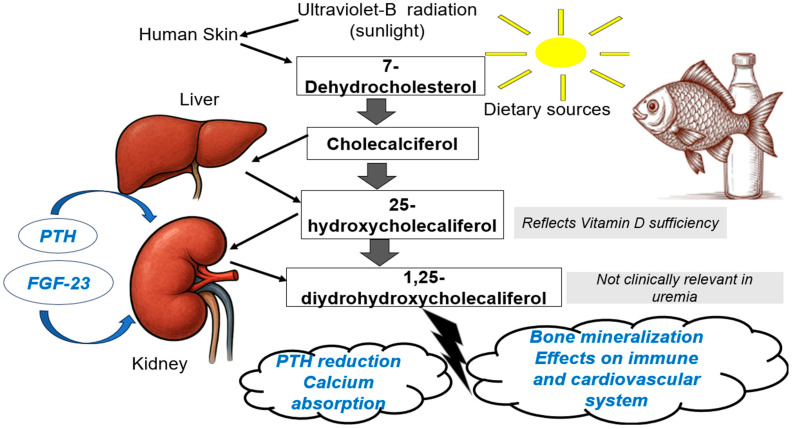
Vitamin D metabolism and functions. FGF-23: fibroblast growth factor 23, PTH: parathormone.

**Figure 2:**
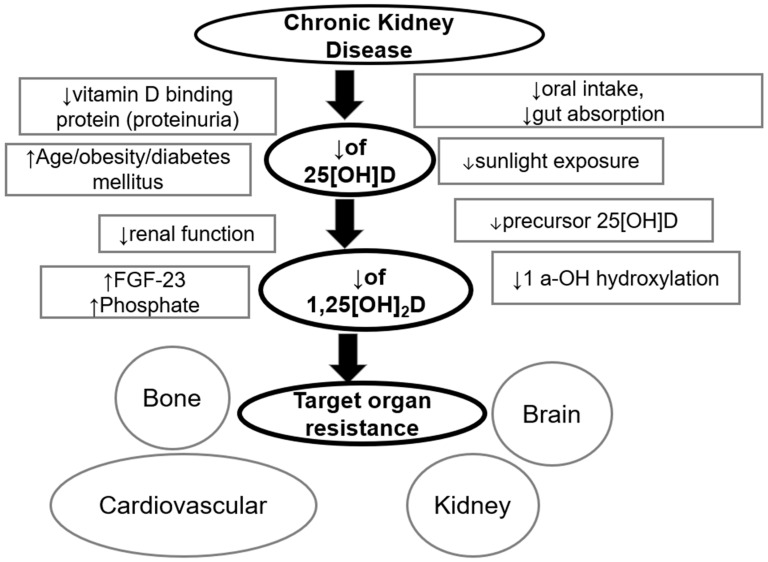
Risk factors for suboptimal vitamin D status in chronic kidney disease patients [31]. ↓ = decreased, ↑ = increased.

CKD patients are prone to developing low levels of vitamin D (Figure 1) due to several reasons, including Glomerular Filtration Rate (GFR) decline, strict dietary restrictions, inadequate exposure to the sun, loss of binding protein in the urine, decreased intrarenal 1α-hydroxylase activity, decrease of megaline, which leads to decreased tubular absorption of 25(OH)D, and uremia and acidosis, which can cause decreased activity of 1a hydroxylase [20,21,22]. Moreover, hypocalcemia and hyperphosphatemia, which develop in CKD and are exacerbated in ESKD, can trigger secondary hyperparathyroidism and upregulate FGF23 [23,24]. Subsequently, PTH and FGF23 interfere with the regulation of 1α-hydroxylase; PTH upregulates its expression to maintain calcium concentrations, and FGF23, driven by phosphate, suppresses the expression of CYP27B1 [25]. Other factors involved in CKD-derived vitamin D deficiency include resistance to vitamin D, reduced hepatic synthesis of CYP24A1 [26], and upregulation of 24–25(OH)2D [21], which is a metabolite produced by increased action of the catabolic enzyme CYP24A1, which causes hydroxylation of 25 (OH)D. Although this metabolite is considered to be inactive, its role is to catabolize 25(OH)D and downregulate 1,25(OH)2D, and thus it might be involved in bone mineral metabolism and calcium/phosphate homeostasis. It has been reported that the 24–25(OH)2D metabolite might also be of benefit in the process of fracture healing. Finally, other factors that predispose to vitamin D deficiency and are not specific to CKD include old age, obesity, diabetes, and hypertension [20,27]. Figure 1 shows the steps of vitamin D activation and functions, whereas Figure 2 shows the risk factors for suboptimal vitamin D status in CKD patients.

It should be noted, however, that vitamin D levels and CKD-BMD parameters might vary significantly among different ethnic groups. A multiethnic study enrolled three different ethnicities and showed that anemia and vitamin D deficiency were more pronounced in black Saharan subjects (compared to Mediterranean and sub-Saharan subjects), and these two entities were interrelated only in black CKD patients [28]. Vitamin D indices might vary significantly in dialysis patients compared to healthy subjects. D3 levels were significantly lower and D2 higher in dialysis compared to controls [29]. KDIGO (Kidney Disease Improving Global Outcomes) guidelines use the same cut-off limits for classification of vitamin D status for patients with CKD and healthy individuals [28]. Insufficient 25(OH)D levels are defined as >10 ng/mL but <20–32 ng/mL (50–80 nmol/L), while 25(OH)D levels < 10 ng/mL (25 nmol/L) constitute deficiency. However, the “normal level” of vitamin D might be the concentration needed to maintain serum PTH levels within normal limits, while others define it as the level of vitamin D at which there is no further reduction in serum PTH with administration of vitamin D supplements [30].

## 3. Discussion

### 3.1. Association Between Vitamin D Deficiency and Adverse Outcomes in Dialysis Patients

Low levels of 25(OH)D have been correlated with CKD–Mineral Bone Disease (CKD-MBD), which is a systemic disorder causing pathological levels of phosphorous, calcium and parathyroid hormone, bones abnormalities, and vascular calcification. CKD-MBD leads to osteopenia, fragile bones, increased risk of fractures, and muscle debility, as well as accelerated vascular calcification and stiffness and CVD [32,33,34,35,36]. Vitamin D is a crucial player in the pathogenesis of CKD-MBD. Vitamin D levels decrease when kidney function fails, while the majority of patients on dialysis exhibit vitamin D deficiency. In addition to the effect of vitamin D on CKD-MBD, accumulating data suggest that vitamin D insufficiency may play a crucial role in the extremely high risk of mortality in patients on dialysis [37,38,39,40]. Vitamin D may also play a protective role against CVD, mental health impairment, infections, cancer, and metabolic syndrome [38,41,42,43,44,45]. In a multicenter, multiethnic, cross-sectional study, Mahdavi et al. enrolled 81 patients undergoing maintenance HD and found that 25(OH)D deficiency was associated with a five-fold increased risk for weak handgrip, a measure of muscle strength and physical condition. Both vitamin D levels and supplementation were correlated with handgrip strength. Notably, the population of this study was much younger than the typical population of a dialysis unit (mean age 58 years), and the study was restricted only to Canada [46]. Studies have shown that in PD patients, deficiency of vitamin D levels has been correlated with cognitive impairment [44], whereas in hemodialysis patients, low levels of vitamin D have been correlated with atherosclerotic disease, insulin resistance, ventricular hypertrophy, [47], vascular calcifications [43], metabolic syndrome, and obesity [44]. Even in children with CKD, vitamin D deficiency has been associated with increased office systolic BP; however, no correlation was found with ambulatory BP measurements [48]. In contrast, a cross-sectional sub-analysis of the IMPROVE-CKD (the IMpact of Phosphate Reduction On Vascular Endpoints in CKD) RCT showed that in 208 patients with CKD stages 3b-4, baseline 25(OH)D levels failed to show any association with surrogate markers of arterial calcification and stiffness, such as pulse wave velocity, augmentation index, abdominal aortic calcification, and lumbar spine BMD [49]. Moreover, it has been reported that marked vitamin D deficiency < 10 ng/mL conferred an increased risk of cognitive impairment and mortality in a large cohort of 17,545 CKD patients followed for 3 years. The limitations of the study included the retrospective design and the fact that the results were derived from a sensitivity sub-analysis; initially, vitamin D deficiency (<20 ng/mL) showed no association with the occurrence of mild cognitive impairment [50]. Furthermore, large studies of CKD patients have shown that vitamin D deficiency is strongly associated with increased risk for developing depression [51]. However, to determine whether restoration of vitamin D levels might protect against the occurrence or even the progression of depression, we need large, well-designed RCTs with large sample sizes and long follow-up periods. In ESKD, vitamin D plays a major role in immunity. This role was brought to the center of scientific research during the COVID-19 pandemic. In HD patients, vitamin D deficiency is associated with increased risk for severe COVID-19 infection and subsequent mortality with a particularly high odds ratio (OR = 22.57, *p* = 0.01 and OR = 15.8, *p* = 0.03, respectively) [52]. Moreover, treatment with an active vitamin D analog was associated with maintenance of high IgG SARS-CoV-2 levels 3 months after vaccination [53]. Ravani et al. reported that 25(OH)D was an independent predictor of disease progression and death in patients with stage 2–5 CKD, even after adjusting for age, heart failure, smoking, C-reactive protein, albumin, phosphate, use of converting enzyme inhibitors or angiotensin receptor blockers, and eGFR [41]. London et al. reported that nutritional vitamin D deficiency and low 1,25(OH)2D3 were risk factors for endothelial dysfunction and arteriosclerosis in HD patients [54].

### 3.2. Kidney Disease Improving Global Outcomes (KDIGO) Guidelines and Vitamin D

Although vitamin D deficiency is independently associated with morbidity and mortality among healthy individuals, restoration of its levels failed to improve any clinical outcomes in dialysis patients. The latest revised KDIGO guidelines for the management of CKD-MBD suggest regular laboratory monitoring of phosphorus, calcium, PTH, and alkaline phosphatase starting from stage 3a (1C). The frequency of monitoring of the forementioned parameters in patients with CKD G3a–G5D varies and depends on the existence and extent of abnormalities and the degree of kidney damage.

Specifically, KDIGO recommend in patients with CKD G3a–G5D that 25(OH)D (calcidiol) levels should be measured frequently, with repeated testing determined by baseline values and therapeutic interventions (2C). Vitamin D deficiency and/or insufficiency should be corrected with the same therapeutic strategies applied to the general population (2C). Therefore, monitoring of 25(OH)D levels should start at stage 3, and, in stage 5D, it should be done at baseline and every 1 year or 6 months if the patient is treated with vitamin D. According to KDIGO guidelines, in HD patients, vitamin D deficiency or insufficiency should be corrected with therapeutic strategies similar to those used for the general population. If 25(OH)D levels are below or equal to 5 ng/mL (severe deficiency), patients should be administered ergocalciferol 50,000 IU/week per os for 12 weeks and then 50,000 IU per month for another 6 months. Levels of 25(OH)D between 5 and 15 ng/mL correspond to mild deficiency and should be treated with ergocalciferol 50,000 IU/week per os for 4 weeks and then 50,000 IU per month for another 6 months. Finally, an HD patient with 25(OH)D levels above 15 but below 30 ng/mL (insufficiency) should be supplemented with ergocalciferol 50,000 IU per month for 6 months. In these cases, vitamin D levels should be monitored every 6 months. Guidelines are grade “C” with “Low” quality of evidence, meaning that the true effect might be substantially different from the estimate of the effect. Because the real target of CKD-MBD in ESKD patients is PTH and not vitamin D levels, KDIGO does not recommend replenishing vitamin D levels but rather reducing ΡΤH levels. Calcimimetics, calcitriol, or vitamin D analogs or a combination of calcimimetics with calcitriol or vitamin D analogs can be used in any order (2B) [5]. Although current nephrology guidelines suggest supplementation of native vitamin D in CKD, similar to the general population, recent data from large RCTs showed no impact of vitamin D on skeletal and non-skeletal outcomes in the general population. Therefore, a very recent, joint European consensus statement by several experts and organizations recommended for CKD patients to screen and correct vitamin D deficiency with a target of 25(OH)D levels over 30 ng/mL and avoid supraphysiological doses over 100,000 IU and markedly increased 25(OH)D levels over 60 ng/mL to avoid toxicity [55]. Real-world data show that 72% of dialysis patients might exhibit vitamin D insufficiency or deficiency. Holden et al. treated 502 maintenance dialysis patients with low vitamin D levels with 50,000 IU/week of cholecalciferol until sufficiency, followed by 50,000 IU/month for maintenance, and found that a 25 nmol/L increase in 25(OH)D levels was accompanied by suppression of PTH, ALP, and phosphate and higher serum calcium [56]. Notably, the authors set a very broad range of vitamin D target values of 30–100 ng/mL. The optimal and exact target value of vitamin D levels in CKD and ESKD remains debatable; guidelines suggest administering vitamin D until reaching levels over 30 ng/mL, but dialysis patients might be prone to vitamin D toxicity due to diminished kidney filtration. To investigate this area of debate, a pilot study in dialysis patients showed that extended-release calcifediol at a very high dose (36,000 IU/week) for 26 weeks was safe and successful in restoring vitamin D levels, suppressing PTH levels, and maintaining circulating calcium and phosphate levels within normal values [57].

### 3.3. Native Vitamin D and Dialysis Patients

Although there are many studies demonstrating the beneficial effect of vitamin D on various organs and tissues, it remains controversial whether native vitamin D supplementation in dialysis patients offers any clinical benefit. Current guidelines suggest that native vitamin D administration should be reserved only for correcting D deficiency and/or insufficiency [21]. Saab et al. showed that most patients on dialysis have vitamin D deficiency and that administering ergocalciferol at a dose of 50,000 IU monthly is a safe and efficacious treatment for restoring vitamin levels [58]. Similar results were reported by Massart et al. for weekly oral administration of 25,000 IU of cholecalciferol for 13 weeks [59]. Delanaye et al. also showed that cholecalciferol administration has similar results and does not negatively affect calcium, phosphorus, PTH levels, or the presence of vascular calcifications [60]. Seven double-blind, randomized, controlled trials evaluated placebo versus natural vitamin D in adult patients undergoing hemodialysis. Zitt et al. showed no significant difference in the increase in serum calcium and phosphorus levels during the 26-week treatment study period, but there was only a decrease in serum PTH. However, this decrease could be attributed to the fact that at baseline some patients were already treated with cinacalcet and calcitriol [61]. A recent RCT in CKD patients with SHPT treated with extended-release calcifediol (ERC) reported that adjunctive active vitamin D further reduced PTH levels by 35% but caused hypercalcemia and impaired kidney function [62]. Bhan et al. failed to show statistically significant differences in serum calcium, phosphorus, and PTH levels between groups in one of the largest clinical trials, which included 105 patients divided into three groups (placebo versus low-dose vitamin D administered weekly and monthly) [63]. Likewise, relatively lower vitamin D doses for 15 weeks were also accompanied by no significant difference in serum phosphorus, calcium, or PTH [64]. All of these studies found that this strategy is effective in restoring vitamin D levels but not in controlling SHPT (contrary to CKD 1–4). Well-designed, statistically powerful, long-term clinical trials are needed to evaluate the impact of nutritional vitamin D on vascular calcification, mortality, and other clinical endpoints [65,66,67,68,69].

Table 1 shows the effect of different treatment strategies using native vitamin D supplementation in HD patients. Vitamin D restored 25(OH)D levels but caused no significant difference in the levels of calcium, phosphate, or PTH.

Moreover, Pikley et al. performed a meta-analysis of 23 trials and 2489 HD patients and found that although vitamin D administration successfully corrects vitamin D deficiency and/or insufficiency, vitamin D has minimal or no effects on various clinical outcomes, including inflammation, nutrition, muscle strength and function, quality of life, hospitalizations, anemia, maturation of arteriovenous fistulae, cardiovascular disease, and overall mortality [69]. A recent retrospective study of 29,654 CKD patients showed that vitamin D deficiency was strongly associated with a more than two-fold increased risk for major adverse renal events, all-cause mortality, and hospitalizations (HR, 2.24, 95% CI, 2.08–2.41; *p* < 0.001, HR, 1.92, 95% CI, 1.82–2.02; *p* < 0.001, and 1.19, 95% CI, 1.14–1.25; *p* < 0.001, respectively) [75], whereas in dialysis patients with SHP receiving calcimimetics, vitamin D deficiency was a strong, independent predictor of 3-year all-cause mortality (HR 1.29, 95% CI: 1.10–1.51, *p* = 0.002) but failed to correlate with hypocalcemia, fractures, and CVD events [76]. Moreover, abnormal lipid metabolism, obesity, and depression increased the effect of the association between vitamin D levels and mortality. Data from a recent meta-analysis including 21 RCTs and 4653 HD patients with SHPT showed that calcimimetics outranked vitamin D analogues in decreasing the calcium–phosphorus product; however, these agents presented significantly more gastrointestinal side effects. Both active D analogues and calcimimetics were successful in reducing serum PTH levels [77].

Similarly, to evaluate the effects of vitamin D compounds on multiple outcomes in CKD patients 3-5D, Yeung et al. conducted a meta-analysis of RCTs [78]. Although not restricted only to dialysis patients, the authors included 7242 dialysis patients and found that treatment with vitamin D reduced PTH levels but increased serum calcium. No effects were found on any of the multiple outcomes, including fractures, all-cause/CVD mortality, CVD events, and hospitalizations. Notably, the quality of the data included was suboptimal due to a small sample size and short-term follow-up periods; moreover, there was significant heterogeneity among the studies included.

Vitamin D is an important element for bone and vascular health and for the management of CKD-MBD. However, the role and clinical effects of vitamin D are not the same in predialysis CKD and ESKD patients [79]. The main forms used for the treatment of SHPT are calcitriol and synthetic vitamin D analogs. Calcitriol (per os or intravenous) and synthetic vitamin D analogs are efficient in reducing serum levels of PTH [39,80,81] by suppressing PTH production, but when PTH is very high, a combination of agents is needed [82,83]. When patients are treated with calcitriol or synthetic vitamin D analogs, caution is warranted for potential adverse effects, such as hyperphosphatemia and hypercalcemia, which might trigger vascular calcification [84,85]. All vitamin D analogues are equal regarding mortality, bone pain, avoiding parathyroidectomy, and other clinical endpoints [86,87]. On the other hand, there are some studies that have tried to determine the influence of restoring vitamin D levels on all-cause mortality. Bhan et al. performed a randomized, multicenter, parallel-group, placebo-controlled trial comparing a placebo with two different doses of ergocalciferol in HD patients and found that 1-year mortality did not differ significantly among the three groups, with the monthly ergocalciferol group at 0% (0 of 33), 8.3% (3 of 33) in the weekly ergocalciferol group, and 13.9% (5 of 36) in the placebo group (*p* = 0.08). When the two groups of ergocalciferol were combined, there was not a significant tendency, in an exploratory analysis, at 1 year of administration (hazard ratio, 0.28, 95% CI, 0.07 to 1.19; *p* = 0.07) [63]. Another randomized, placebo-controlled, double-blind trial with 1-year follow-up in dialysis patients with a 25(OH)D concentration < 50 nmol/L showed that compared to placebo, 50.000 IU/week oral cholecalciferol had no effect on muscle strength, blood pressure, bone parameters, levels of calcium, phosphorus, PTH, or cardiac ischemia [88]. In agreement with these results, a multicenter, open-label, randomized trial of 284 HD patients with vitamin D insufficiency showed that calcifediol supplementation for 24 months failed to improve mortality and cardiovascular outcomes [89]. Moreover, a 2 × 2 factorial design randomized controlled trial with Bioelectrical Impedance Analysis (BIA)-guided volume management versus standard care and oral cholecalciferol 50,000 U/week for 8 weeks, followed by 10,000 U/week for another 44 weeks or placebo, showed that vitamin D treatment showed no improvement in LV mass [90]. Likewise, several other studies in dialysis patients failed to demonstrate any positive effect of administration of vitamin D on hospitalizations [63,70,88,89,90] or CVD, cardiac outcomes [63,89,90], left ventricular mass and function, pulse wave velocity (PWV), abdominal aortic calcification (AAC) [60,90,91,92], and congestive heart failure [92,93]. Similarly, an updated meta-analysis in 2024 included 11 RCTs in CKD patients and found that vitamin D treatment failed to show any beneficial effect on cardiovascular events, left ventricular hypertrophy, ejection fraction, and blood pressure [94]. A meta-analysis of 12 trials in CKD patients showed that both paricalcitol and cholecalciferol significantly improved FMD but had no effect on other indices of vascular function, including PWV and Aix [95]. Even in the limited and fragile population of patients at the last stage of CKD who are about to start dialysis and are prone to hypocalcemia, reduced calcium levels were associated with overhydration and worse cardiac function; moreover, the use of vitamin D analogues was a strong and significant effect modifier of these associations. The authors concluded that treatment with vitamin D analogues might improve fluid status and cardiac function in hypocalcemic ESKD patients [96]. Although these studies had significant heterogeneity in terms of doses, routes, study duration, and endpoints, collectively, there are no data supporting any beneficial CV outcome of restoration of vitamin D levels in HD. CKD-MBD mainly affects bones and muscles. Administration of vitamin D supplementation in HD patients does not improve muscle grip [71,88] or inflammation status, assessed by C-reactive protein values [70,71,91,92,97,98,99]. Only in one study was there a statistically significant difference in CRP in patients receiving vitamin D treatment, but the sample size was small and the study period too short to draw any definite conclusions [99]. The Japan Dialysis Outcomes and Practice Patterns Study showed that in 1875 walking HD patients with poor physical condition, compared to treatment with calcimimetics, vitamin D analogues were associated with a significantly reduced risk for falls [100]. Using data from the multiethnic Dialysis Outcomes and Practice Patterns Study (DOPPS) study with 41,677 HD patients from 21 countries, the rates of bone fracture were not different according to vitamin D analogue prescription [101]. Moreover, one of the pleiotropic beneficial effects of vitamin D might also be the correction of renal anemia. A recent RCT showed that in HD patients with anemia and vitamin D deficiency, vitamin D therapy (50,000 IU/month) for 6 months was associated with improved Hb levels and decreased dosages of erythropoietin agents [102]. Although vitamin D is suggested to play a major role in nutritional or metabolic well-being, restoration of vitamin D levels to normal levels did not improve serum albumin levels or other nutrition markers in dialysis populations [90,91,93,97]. During recent years, it has become evident that oxidative stress (OS) and inflammation are triggered by dialysis and affect patients’ outcomes [103]. OS is common in uremic patients and considered a pathogenetic mechanism and a nontraditional risk factor for adverse events, such as all-cause and CV mortality. OS is present from the early stages of CKD, worsens as CKD progresses to ESKD, and is aggravated in patients undergoing dialysis [103]. Vitamin D supplementation may play a role in OS. It is reported that vitamin D administration in diabetic HD patients might improve their metabolic profiles and exert additional antioxidant effects [104,105]. Risk factors that correlate with a uremic milieu and dialysis parameters, e.g., OS, inflammation, and disorders of calcium/phosphorus levels, might trigger the onset of vascular calcification and CVD in ESKD patients, and vitamin D might indirectly prevent VC and CVD. Matrix Gla Protein (MGP), a powerful suppressor of calcification in vivo and in vitro [106], is a vitamin-K-dependent protein that needs vitamin K to become biologically active. It has been suggested that there may be a positive interaction between vitamins D and K and impacts on the skeleton and the CV. Genetic, molecular, and cellular studies on experimental animals and humans indicate that most effective concentrations of both of the vitamins (D and K) are favorable for the skeleton and CV [107]. RCTs showing the effects of vitamin D/VDRAs in HD patients according to different systems are shown in Table 2.

## 4. Materials and Methods

This is a narrative review assessing observational and clinical studies regarding vitamin D and vitamin D analogues in CKD and ESKD. Our search strategy included the keywords “vitamin D,” “CKD,” “ESKD,” and “Hemodialysis” in Medline/PubMed from 2001 to 2025 in English. We assessed the studies that KDIGO guidelines are based on and expanded our literature search to clinical studies in HD populations. Moreover, we included recent meta-analyses in this area and KDIGO guidelines and consensus statements.

## 5. Conclusions

Vitamin D and vitamin D analogues supplementation succeed as a treatment at increasing 25(OH)D and managing CKD-MBD without severe episodes of hypercalcemia or vitamin D toxicity but fail to show any benefit in dialysis patients with regard to clinical hard outcomes, such as cardiovascular mortality, morbidity, cognitive impairment, inflammation, and mortality. Currently, the guidelines suggest supplementation of native vitamin D only to correct insufficiency or deficiency. However, the real target in ESKD is PTH; in this scenario, vitamin D, vitamin D analogues, and calcimimetics can be used in any order or combination. This is completely different from CKD stages 1–4, where vitamin D might reduce PTH levels successfully. Therefore, vitamin D metabolism, monitoring, targets, and treatment strategies differ significantly from the general population to predialysis CKD and ESKD.

In dialysis patients, existing data on vitamin D and vitamin D analogues are conflicting and derived from small studies that are mainly observational, with large heterogeneity, poor design, and short follow-up. The few RCTs conducted so far failed to consistently show benefits of vitamin D or vitamin D analogues on clinical hard outcomes. Based on current guidelines and the recent European consensus statement, in CKD, vitamin D deficiency should be monitored and corrected until 25(OH)D levels reach 30 ng/mL; caution is warranted for high doses of vitamin D supplementation and 25(OH)D levels over 60 ng/mL. The most interesting results so far were obtained during the COVID-19 pandemic regarding the potential beneficial effects of vitamin D (both native and active) on the severity of respiratory infections and stabilization of antibodies after vaccination. However, these data are also derived from observational and retrospective studies. The major limitation of this paper is that it is a narrative review and not a systematic review with a structured methodology and design; therefore, the conclusions reported should be considered with caution.

However, the most recent systematic reviews and meta-analyses regarding vitamin D in dialysis [69,78] were inconclusive and highlighted the fact that the studies so far have suboptimal quality, small sample sizes, and short durations and suffer from significant heterogeneity. In this area, we need large, well-designed, prospective RCTs with multiple and clinical hard outcomes to determine when, how much, and for how long treatment should be administered and what type of vitamin D is needed for the treatment of dialysis patients and to complete the unfinished tale of the role of vitamin D and vitamin D analogues in dialysis.

## Figures and Tables

**Table 1:** RCTs showing the effects of native vitamin D supplementation in HD patients in relation to CKD-MBD parameters.

Study/Ref.	Population	Treatment	Study Period	Outcome	Result
Zitt et al. [61]	56 HD patients with 25(OH)D < 20 ng/mL	100 IU p.o cholecalciferol/kg body weight 1/week	26 weeks	Difference in 25(OH)D3 Ca P PTH	Increase (*p* = 0.01) No difference No difference Decrease (*p* = 0.01)
Bhan et al. [63]	105 HD patients with 25(OH)D levels ≤ 32 ng/mL	50,000 IU po ergocalciferol 1/week or 1/month or placebo	12 weeks	Difference in 25(OH)D3 Ca P PTH	Increase (*p* = 0.001) No difference No difference No difference
Armas et al. [64]	42 HD patients with 25(OH)D < 16.2 ng/mL	10,133 IU p.o cholecalciferol 1/week	15 weeks	Difference in 25(OH)D3 Ca P PTH	Increase (*p* < 0.001) No difference No difference No difference
Kooienga et al. [65]	610 elderly women with predialysis CKD and 25(OH)D < 15 ng/mL	1200 mg tricalcium phosphate and 800 IU cholecalciferol	24 months	Difference in 25(OH)D3 Ca P PTH	Increase No difference No difference Decrease
Marckmann et al. [67]	25 CKD patients and 27 HD patients with 25(OH)D < 50 nmol/L	40,000 IU p.o cholecalciferol 1/week	8 weeks	Difference in 25(OH)D3 Ca P PTH Difference in 25(OH)D3 Ca P PTH	CKD Increase (*p* < 0.001) No difference No difference Decrease (*p* < 0.001) HD Increase (*p* < 0.001) No difference No difference No difference
Wasse et al. [68]	52 HD patients with 25(OH)D < 25.5 ng/mL	200,000 IU p.o cholecalciferol 1/wk	3 weeks	Difference in 25(OH)D3 Ca P PTH	Increase (*p* < 0.001) No difference No difference No difference
Miskulin et al. [70]	276 HD patients with 25(OH)D < 22.6 ng/mL	p.o ergocalciferol 50,000 IU 1/week for 6 months for patients with 25(OH)D ≤ 15 ng/m, 50,000 IU 1/week for the first 3 months followed by 50,000 IU 1/month for another 3 months when 25(OH)D was between 16 and 30 ng/mL	6 months	Difference in 25(OH)D3 Ca P PTH	Increase (*p* < 0.001) No difference No difference No difference
Hewit et al. [71]	60 HD patients with 25(OH)D ≤ 24 ng/mL	50,000 IU p.o cholecalciferol 1/week for 8 weeks and then 1/month for 4 months	6 months	Difference in 25(OH)D3 Ca P PTH Episodes of hypercalcemia Episodes of hyperphosphatemia	Increase (*p* < 0.001) No difference Decrease (*p* = 0.03) No difference No difference
Alsahawey et al. [72]	60 HD patients	200,000 IU per os cholecalciferol 1/month	3 months	Difference in 25(OH)D3 Ca P PTH Adverse events	Increase (*p* < 0.001) No difference No difference No difference No difference
Matias et al. [73]	158 HD patients	p.o cholecaliferol 50,000 IU 1/week for patients with 25(OH)D levels < 15 ng/mL, 10,000 IU 1/week when 25(OH)D was between 16 and 30 ng/mL, 2700 IU 3/week when levels were >30 ng/mL	12 months	Difference in 25(OH)D3 Ca P PTH	Increase (*p* < 0.001) Decrease (*p* = 0.014) Decrease (*p* = 0.011) Decrease (*p* < 0.0001)
Tokmak et al. [74]	64 HD patients	20,000 IU cholecalciferol p.o 1/week for 9 months. Followed by 20,000 IU cholecalciferol p.o 1/month for 15 months	24 months	Difference in 25(OH)D3 Ca P PTH	Increase (*p* < 0.001) Increased (*p* < 0.01) No difference No difference

Ca, calcium; CKD, chronic kidney disease; HD, hemodialysis; P, phosphorus; PTH, parathormone.

**Table 2:** RCTs showing the effects of vitamin D/VDRAs in HD patients according to different systems.

Study Ref.	Population	Treatment	Study Period	Outcome	Result
Glycemic and lipid metabolism					
Hung et al. [108]	10 HD treated with paracalcitol	Cinacalcet or restart paracalcitol	8 weeks	GDR HOMA-IR QUICKY	No change No change No change
Hosseini et al. [109]	55 diabetic HD	Vit. D 50,000 IU/15 days vs. placebo	12 weeks	PPAR-γ PI3K IRS1, IRS2 GLUT-4 PKC LDLR, Lp(a) PDK1	↑ expression of PPAR-γ, AKT, PI3K, IRS1, and GLUT4 genes ↓ expression of PKC and LDLR genes No change in PDK1, IRS2, and Lp(a) expression
Tamadon et al. [104]	60 diabetic HD	Vit. D3 50,000 IU/15 days vs. placebo	12 weeks	Insulin concentration HOMA-IR QUICKI Lipid metabolism parameters	↓ insulin ↓ HOMA-IR ↑ QUICKY Lipid metabolism parameters: no change
Anemia					
Emarah et al. [102]	100 anemic HD patients with vitamin D deficiency	Vit. D 50,000 IU monthly vs. placebo	6 months	Markers of anemia management	Ferritin, iron, transferrin saturation: no change ↑ Hb and ↓ EPO dosage
Miskulin et al. [70]	276 HD with serum 25(OH)D < 30 ng/mL	Ergocalciferol vs. placebo	6 months	EPO dosage	No change
Matias et al. [73]	158 HD	Cholecalciferol −50.000 IU 1/week for patients with 25(OH)D < 15 ng/mL −10,000 IU 1/week for 16 < 25(OH)D < 30 ng/mL −2700 IU 3/week for 25(OH)D > 30 ng/mL	12 months	EPO dosage	↓ (*p* = 0.013)
Cardiovascular system and hard endpoints					
Raggi et al. [110]	360 HD with SHPT and CAC scores ≥ 30	Cinacalcet (30–180 mg/day) + low-dose calcitriol vs. flexible vitamin D	52 weeks	Progression of vascular and cardiac valve calcification (% change of CAC score)	Cinacalcet group: slower progression of CAC scores and volume scoring
El Borolossy et al. [111]	60 children HD	100 µg MK-7 vs. 10 µg vit. D vs. 100 µg MK-7+ 10 µg vit. D vs. controls	4 months	Vascular calcification regulators	The group treated with 100 µg MK-7+ 10 µg vit. D showed the most significant ↓ in dp-ucMGP, uc-OC; no change in FGF-23
Hewit et al. [71]	60 HD with 25(OH)D < 24 ng/mL	Cholecalciferol, 50,000 IU/week for 8 weeks followed by 50,000 IU/month for 4 months	6 months	Pulse wave velocity Muscle strength Functional capacity Quality of life	No change No change No change No change
Hansen et al. [112]	57 HD	Paricalcitol vs. alfacalcidol	16 weeks	Vascular calcification regulators	NT-proBNP and osteoprotegerin ↑ in both groups Fetuin-A ↑ significantly in the alfacalcidol-treated group
Miskulin et al. [70]	276 HD with serum 25(OH)D < 30 ng/mL	Ergocalciferol vs. placebo	6 months	All-cause, cardiovascular-related hospitalizations	No change
Bhan et al. [63]	105 HD with 25(OH)D ≤ 32 ng/mL	Ergocalciferol, 50,000 IU/week vs. Ergocalciferol, 50,000 IU/month vs. placebo	12 weeks	All-cause and cause-specific hospitalizations	No change
Mann et al. [113]	56 HD	2 × 2 crossover RCT Intensive (alfacalcidol) 0.25 mcg, thrice weekly + ergocalciferol 50.000 IU/week vs. standard (alfacalcidol) 0.25 mcg thrice weekly for 6 weeks	6 weeks	Cardiac autonomic tone low frequency to high-frequency spectral ratio	↑ low-frequency to high-frequency spectral ratio only in patients with 25[OH]D < 20 ng/mL)
Immune and endocrine system					
Nata et al. [114]	70 HD with 25[OH]D level < 30 ng/mL	Ergocalciferol Conventional (50.000 IU/month for 25[OH]D between 20 and 29.9 ng/mL and 50.000 IU/week for <20 ng/mL) vs. high dose (100.000 IU/month for 25[OH]D between 20 and 29.9 ng/mL and 100.000 IU/week for <20 ng/mL)	8 weeks	IL-6	In patients with 25[OH]D < 20 ng/mL, high-dose treatment ↓ serum IL-6 level (−2.67 pg/mL [IQR −6.56 to −0.17], *p* = 0.039)
Gregorio et al. [91]	32 HD	Cholecalciferol vs. placebo	6 months	Circulating IL-1β and hs-CRP levels In vitro OS markers (monocyte viability, ROS production, and CAMP expression)	Circulating IL-1b and hs-CRP: no change ↓ all OS markers
Meireles et al. [98]	38 HD with 25(OH)D < 20 ng/mL	Cholecalciferol group 50,000 IU/twice weekly vs. placebo	12 weeks	Expression of VDR, CYP27B1, CYP24A1, and IL-6 in monocytes; serum concentrations of IL-6, TNF-α, CRP	↑ CYP27B1 ↑ VDR expression No changes in IL-6 and CYP24A1 ↓ serum concentration of IL-6 and CRP
Hansen et al. [112]	57 HD	Paricalcitol vs. alfacalcidol	16 weeks	IL-6 TNF- α hs-CRP	No change
Tamadon et al. [104]	60 diabetic HD	Vit. D3 50,000 IU/15 days vs. placebo	12 weeks	Hs-CRP MDA TAC	↓ Hs-CRP ↓ MDA ↑ TAC
Zheng et al. [115]	60 HD with SHPT (PTH > 300 pg/mL) receiving 2 mcg/day of paricalcitol	Cholecalciferol 5000 IU/week vs. placebo	16 weeks	hCAP-18	↑
Miskulin et al. [70]	276 HD with serum 25(OH)D < 30 ng/mL	Ergocalciferol vs. placebo	6 months	CRP Infection-related hospitalizations	No change No change
Matias et al. [73]	158 HD	Cholecalciferol −50.000 IU 1/week for patients with 25(OH)D < 15 ng/mL −10,000 IU 1/week for 16 < 25(OH)D < 30 ng/mL −2700 IU 3/week for 25(OH)D > 30 ng/mL	12 months	CRP	↓ (*p* = 0.004)
Hung et al. [108]	10 HD treated with paracalcitol	Cinacalcet or restart paracalcitol	8 weeks	Hs-CRP IL-6 Adiponectin and leptin	No change No change No change
Ulrich et al. [116]	33 HD	Cholecalciferol vs. placebo	12 weeks	Serum testosterone levels	No change

↓: decreased; ↑: increased; CAC: coronary arterial calcification; CAMP: cyclic adenosine monophosphate; Dp-ucMGP: dephosphorylated, uncarboxylated Matrix Gla Protein; EPO: erythropoietin; FGF-23: fibroblast growth factor 23; GDR: glucose disposal rate; GLUT-4: glucose transporter type 4; Hb: hemoglobin; HD: hemodialysis; HOMA-IR, homeostasis model of assessment–estimated insulin resistance; HCAP-18: human cathelicidin; IL: interleukin; IRS1: insulin receptor substrate-1; Lp(a): lipoprotein (a); LDLR: low-density lipoprotein receptor; MDA: malondialdehyde; MK-7: menaquinone-7; NT-proBNP: N-terminal pro b-type natriuretic peptide; OS: oxidative stress; PI3K: phosphatidylinositol 3-kinase; PDK1: pyruvate dehydrogenase kinase 1; PKC: protein kinase C; PPAR-γ: proliferation-activated receptor gamma; PTH, parathormone; QUICKY: quantitative insulin sensitivity check index; ROS: reactive oxygen species; SHPT: secondary hyperparathyroidism; TAC: total antioxidant capacity; TNF-α: tumor necrosis factor-α; Uc-OC, uncarboxylated osteocalcin; VDR: vitamin D receptor.

## Data Availability

No new data were created or analyzed in this study. Data sharing is not applicable to this article.
